# Inverse Correlation Between Bone Mineral Density and Systemic Immune Inflammation Index in Postmenopausal Turkish Women

**DOI:** 10.7759/cureus.37463

**Published:** 2023-04-12

**Authors:** Hakan Yolaçan, Serkan Guler

**Affiliations:** 1 Orthopaedics and Traumatology, Aksaray University Training and Research Hospital, Aksaray, TUR

**Keywords:** systemic immune inflammation index, menopause, osteopenia, bone mineral density, postmenopausal osteoporosis

## Abstract

Aim: In this study, the correlation between the change in bone mineral density (BMD) and the numerical value of the systemic immune inflammation index (SII) in postmenopausal Turkish women was investigated.

Material and methods: The study comprised patients who had undergone menopause for at least one year and whose blood samples were examined by dual-energy X-ray absorptiometry (DEXA) on the same day. A retrospective evaluation of 527 postmenopausal women was performed. Age, body mass index (BMI), BMD, *t* score, neutrophil-lymphocyte-platelet-monocyte counts, neutrophil-to-lymphocyte ratio (NLR), platelet-to-lymphocyte ratio (PLR), monocyte-to-lymphocyte ratio (MLR), and SII (platelet count x neutrophil count/lymphocyte count) values were calculated and recorded.

Results: When the data were analyzed with the Statistical Package for the Social Sciences (SPSS; IBM SPSS Statistics for Windows, version 26.0. Armonk, NY: IBM Corp.), neutrophil (p<0.001), lymphocyte (p=0.004), monocytes (p=0.002), platelet distribution width (PDW) (p=0.02), procalcitonin (PCT) (p=0.04), erythrocyte sedimentation rate (ESR) (p=0.001), C-reactive protein (CRP) (p=0.001), NLR (p=0.001), PLR (p=0.004), MLR (p=0.003), and SII (p=0.001) values were found to be higher in the postmenopausal osteoporosis (PMO) group than in the normal and osteopenia groups (p<0.05). When the platelet (p=0.15), mean platelet volume (MPV) (p=0.07), and 25-OH vitamin-D (p=0.15) values were compared, no correlation was found between the groups (normal, osteopenia, and PMO) (p>0.05).

Conclusion: This study revealed that NLR, PLR, MLR, and SII values were inversely correlated with the change in BMD in postmenopausal Turkish women. These biomarkers, which are obtained from a simple, affordable, and accessible routine blood examination, may play an effective role in the early diagnosis and prevention of PMO in the future.

## Introduction

Postmenopausal osteoporosis (PMO) is a chronic, systemic disease characterized by bone loss and impaired bone metabolism and microstructure, increasing the fragility of bone. Fractures due to osteoporosis are difficult to treat and have enormous economic consequences. In addition, postmenopausal fractures reduce daily activities, resulting in a decrease in their quality of life. Accordingly, there is an increase in the frequency of cardiopulmonary dysfunction and venous thrombosis. Estrogen deficiency, advanced age, tobacco use, and a sedentary lifestyle are risk factors for osteoporosis [1,2].

Osteoporosis is a global problem. As a result of low bone density due to osteoporosis, fractures occur most frequently in the proximal femur, vertebrae, and distal radius. In addition, these fractures are associated with high mortality and morbidity. Rapid population growth causes an increase in the number of osteoporosis cases. This has a negative impact on social and economic well-being and causes significant health problems [3,4].

One of the most effective methods for managing PMO treatment is early detection; therefore, it is important to identify simple, accessible, and cost-effective biomarkers [5]. It is known that the pathogenesis of PMO is associated with immune system dysfunction and systemic inflammation [6,7]. Therefore, a non-invasive and objective diagnostic test is necessary at the initial stage of the disease.

When the latest data are evaluated, it is seen that inflammation has critical importance in the process of bone remodeling and osteoporosis. Bone immunology studies the effect of immune system elements on the development of osteoporosis. The neutrophil-to-lymphocyte ratio (NLR) and platelet-to-lymphocyte ratio (PLR) in the peripheral blood samples are newly proposed inflammatory markers with the advantages of being easy, cost-effective, and non-invasive [8]. However, the relationship between NLR, PLR, MLR, and PMO has not yet been fully established.

In this study, the relationship between bone mineral density (BMD) and systemic immune inflammation index (SII) in postmenopausal Turkish women was investigated.

The hypothesis of our study is that SII values are higher in people with PMO and are inversely proportional to BMD.

## Materials and methods

This study was approved by the Ethics Committee of Aksaray University Training and Research Hospital in 2022 with decision number 11-01. In our study, patients who were registered in the archives of our hospital between January 1, 2021 and January 1, 2022, who had dual-energy X-ray absorptiometry (DEXA) examination on the same day and whose peripheral blood samples were taken, and who had a history of menopause for at least one year were included in the study. To obtain a comprehensive blood routine examination, venous blood samples of about 6 mL were collected from all participants after overnight fasting. Then, the blood samples were soon sent to the department of the clinical laboratory in our hospital and tested by an automatic blood cell analyzer. The study excluded patients who had menopause for less than one year, a history of acute or chronic infection, liver or kidney disease, rheumatological disease, autoimmune disease, cancer, hematological disease, steroid use, and blood transfusion within the last previous year. A retrospective evaluation of 527 patients was performed. Age, body mass index (BMI), BMD (femoral neck), BMD (L2-L4), t score (femoral neck), t score (L2-L4), neutrophil-lymphocyte-platelet-monocyte counts, platelet distribution width (PDW), mean platelet volume (MPV), procalcitonin (PCT), erythrocyte sedimentation rate (ESR), C-reactive protein (CRP), and 25-OH vitamin-D values were recorded. NLR, PLR, MLR, and SII (platelet count x neutrophil count/lymphocyte count) [9] values were calculated and recorded.

BMD was measured by DEXA in postmenopausal women and the patients were divided into three groups. World Health Organization (WHO) classifies individuals with a t score of less than or equal to −2.5 as having PMO, individuals with a t score of less than −1 as having osteopenia, and individuals with a t score of more than −1 as having normal bone [10].

Statistical analysis

We evaluated the obtained data with the Statistical Package for the Social Sciences (SPSS) version 26.0 for Windows (Armonk, NY: IBM Corp.). We used the Kolmogorov-Smirnov test to evaluate whether the data were homogeneous. Groups were compared with a one-way analysis of variance (ANOVA). Pearson's correlation coefficient was determined and the correlation between the variables was evaluated. A p-value less than 0.05 was considered significant.

## Results

All variables were analyzed for 527 individuals evaluated in the study and the individuals were divided into three groups (normal, osteopenia, and PMO). When all three groups were evaluated in terms of age (p=0.54) and BMI (p=0.18), no correlation was found (p>0.05) (Table 1). On the other hand, when we look at the BMD (femur neck) (p<0.001), BMD (L2-L4) (p<0.001), t score (femur neck) (p<0.001), and t score (L2-L4) (p<0.001) variables, these values were found to be lower in the PMO group (p<0.05) (Table 1). Similarly, a significant difference was found between the groups in terms of neutrophil (p<0.001), lymphocyte (p=0.004), monocytes (p=0.002), PDW (p=0.02), PCT (p=0.04), ESR (p=0.001), CRP (p=0.001), NLR (p=0.001), PLR (p=0.004), MLR (p=0.003), and SII (p=0.001) values, and these values were higher in the PMO group (p<0.05). On the other hand, when the platelet (p=0.15), MPV (p=0.07), and 25-OH vitamin-D (p=0.15) values were examined, no difference was found between the groups (p>0.05) (Table 1).

**Table 1:** The mean and standard deviation values of the variables for the normal, osteopenia, and PMO groups BMI: body mass index; BMD: bone mineral density; PDW: platelet distribution width; MPV: mean platelet volume; PCT: procalcitonin; ESR: erythrocyte sedimentation rate; CRP: C-reactive protein; NLR: neutrophil-to-lymphocyte ratio; PLR: platelet-to-lymphocyte ratio; MLR: monocyte-to-lymphocyte ratio; SII: systemic immune inflammation index; PMO: postmenopausal osteoporosis.

	Normal (n=105)	Osteopenia (n=274)	PMO (n=148)	p-Value
Age (years)	51.6±7.3	53.4±9.4	54.1±8.5	0.54
BMI	29.6±4.3	29.9±4.8	29.4±3.8	0.18
BMD (femoral neck)	1.01±0.1	0.82±0.07	0.75±0.09	<0.001
BMD (L2-L4)	1.17±0.07	1.07±0.09	0.77±0.05	<0.001
t-Score (femoral neck)	-0.1±0.7	-1.4±0.4	-2.7±0.1	<0.001
t-Score (L2-L4)	-0.1±0.5	-1.2±0.1	-3.1±0.5	<0.001
Neutrophil	3.44±0.73	4.32±1.33	4.54±1.44	0.001
Lymphocyte	1.9±0.34	2.13±0.4	2.65±1.06	0.004
Monocyte	0.36±0.07	0.39±0.05	0.43±0.15	0.002
Platelet	279±76	286±59	278±96	0.15
PDW	16.09±0.45	16.17±0.34	16.27±0.38	0.02
MPV	9.78±1.31	10.11±1.02	10.06±1.51	0.07
PCT	0.26±0.04	0.28±0.04	0.29±0.05	0.04
ESR	13.1±6.4	17.1±11.6	17.3±9.2	0.001
CRP	1.8±1.02	2.5±1.56	4.2±2.3	0.001
25-OH vitamin-D	18.3±7.5	17.4±6.7	19.9±8.4	0.15
NLR	1.64±0.34	1.87±0.76	2.3±0.81	0.001
PLR	134.3±34	138.2±36.6	149.9±54.9	0.004
MLR	0.14±0.03	0.17±0.06	0.19±0.03	0.003
SII	456.6±145.7	530.7±225.8	642.1±362.7	0.001

## Discussion

PMO is an important public health problem due to the high mortality and morbidity it causes. It is also an economic problem due to the resulting loss of the workforce and increased healthcare expenditures. The fact that it is preventable causes a continuous search for early detection methods. We hypothesized that early diagnosis of PMO is possible through routine biochemical analysis, which is an easily available and relatively inexpensive method.

In our study, there was no difference between the groups in terms of age and BMI variables. This shows that the groups for these two variables are homogeneously distributed and do not affect the results. Similarly, no effect of platelet count, MPV, and 25-OH vitamin-D levels on BMD values and t-scores was found.

Neutrophil-lymphocyte-monocyte counts and PDW, PCT, ESR, and CRP levels were inversely correlated with BMD values and t-scores and were significantly higher in the PMO group. It was also shown that NLR, PLR, MLR, and SII values increased as the BMD value and t-score decreased.

It has been suggested that there is a relationship between inflammation, osteoporosis, rheumatoid arthritis, and inflammatory bowel disease. It is stated that bone metabolism is negatively affected in inflammatory diseases, such as systemic lupus erythematosus [11]. Another study revealed that inflammatory cytokines affect bone metabolism via the RANK/RANKL/OPG pathway [12].

Eroğlu and Karataş [13] found that PLR was higher and vitamin-D level was lower in a study they conducted with the PMO group. Gao et al. [14] found that the MLR, NLR, and PLR values ​​of patients with osteoporosis were significantly higher than the control group in a study they conducted with MLR, and this result was found to be similar to our study. Öztürk et al. [15] found that the NLR value was significantly higher in the osteoporotic fracture group. In the study of Köseoğlu [16], it was shown that BMD values ​​were inversely proportional to PLR, but there was no significant relationship with NLR. In our study, in addition to PLR, NLR was also shown to have an inverse relationship with BMD. According to Lee et al.'s [17] study with Korean PMO women, there was a significant relationship between BMD with PLR and NLR, and it was shown that PLR and NLR values ​​increased as BMD decreased, which was consistent with our study. Similarly, Fang et al. [18] reported that in a multicenter and prospective study of BMD, SII, NLR, PLR, MLR, and PDW values ​​were significantly higher in the osteoporotic group and showed an inverse correlation with BMD, and these results were interpreted as being consistent with our study.

Study limitations

The limitations of the study are that it is retrospective, there is no evaluation of alcohol and tobacco use, and there is no discussion of vitamin-D supplementation. Our study is the first study in the literature to examine the relationship between NLR, PLR, MLR, and SII values and PMO in postmenopausal Turkish women. The fact that the number of patients in our study is higher than that in other studies in the literature is the plus of our study.

## Conclusions

PMO is a global public health problem and it is extremely important to reveal biomarkers that can be used in its early diagnosis for its prevention. In addition, the simple, cost-effective, and accessible biomarkers to be used will pave the way for their more widespread use, thus enabling a wider assessment of the population at risk. In this study, cost-effective and easily accessible biomarkers such as NLR, PLR, MLR, and SII, which can be obtained after a routine blood examination, were shown to be inversely related to BMD in postmenopausal Turkish women.
